# A pilot feasibility trial of alcohol screening and brief intervention in the police custody setting (ACCEPT): study protocol for a cluster randomised controlled trial

**DOI:** 10.1186/s40814-015-0001-7

**Published:** 2015-03-03

**Authors:** Jennifer Birch, Stephanie Scott, Dorothy Newbury-Birch, Alan Brennan, Heather Brown, Simon Coulton, Eilish Gilvarry, Matthew Hickman, Elaine McColl, Ruth McGovern, Colin Muirhead, Eileen Kaner

**Affiliations:** 1 Institute of Health and Society, Newcastle University, Baddiley Clark Building, Richardson Road, Newcastle Upon Tyne, NE2 4AX UK; 2 Health Economics and Decision Science, The University of Sheffield, Regent Court, 30 Regent Street, Sheffield, S1 4DA UK; 3 Centre for Health Services Studies, University of Kent, Canterbury, Kent CT2 7NZ7NZ UK; 4 Newcastle & North Tyneside Addictions Service, Plummer Court, Carliol Place, Newcastle upon Tyne, NE1 6UR UK; 5 School of Social and Community Medicine, University of Bristol, Canynge Hall, 39 Whatley Road, Bristol, BS8 2PS UK

**Keywords:** Alcohol, Screening and brief intervention, Feasibility pilot trial, Behaviour change, Police custody

## Abstract

**Background:**

There is evidence of an association between alcohol use and offending behaviour and around a quarter of police time is spent on alcohol-related incidents. Police custody, therefore, provides an important opportunity to intervene. This pilot trial aims to investigate whether a definitive evaluation of screening and brief interventions aimed at reducing risky drinking in arrestees is acceptable and feasible in the custody suite setting.

**Methods:**

Screening will be carried out by trained detention officers or drug and alcohol workers in four police forces across two geographical areas (North East and South West England). Detention officers (or drug and alcohol workers) will be cluster randomised to one of three conditions: screening only (control group), screening followed immediately by 10 min of manualised brief structured advice delivered by the individual responsible for screening (intervention 1) or screening followed by 10 min of manualised brief structured advice delivered by the individual responsible for screening plus the offer of a subsequent 20-min session of behaviour change counselling delivered by a trained alcohol health worker (intervention 2). Participants will be arrestees aged 18+ who screen positive on the Alcohol Use Disorders Identification Test. Participants will be followed up at 6 and 12 months post-intervention. An embedded qualitative process evaluation will explore acceptability of alcohol screening and brief intervention to staff and arrestees as well as facilitators and barriers to the delivery of such approaches in this setting.

**Results:**

Recruitment is currently underway and due to end May 2015.

**Conclusion:**

Results from this pilot trial will determine if a definitive evaluation is possible in the future and will provide stakeholder input to its design.

**Trial registration:**

Reference number: ISRCTN89291046.

## Background

There is plentiful evidence of an association between alcohol use and offending behaviour [1-3]; however, the precise relationship is complex [4,5] due to an intricate interplay between drinking patterns, the amount of alcohol consumed and individual and contextual factors [6]. Alcohol has been found to be a factor in half of all violent crimes (defined as assaults, robbery and snatch thefts where the victim considered the perpetrator to be ‘under the influence’ of alcohol) [7]. In England and Wales, alcohol-related crime is estimated to cost society £11 billion per annum (2010–2011 costs) [8]. Moreover, around a quarter of police time is spent on alcohol-related incidents [9]. Recent research from the North East of England showed that 66% of adult offenders in probation and the prison system [10] and 59% of arrestees in a police custody setting [11] were hazardous, harmful or dependent drinkers. In both studies, screening for problematic drinking was carried out using the Alcohol Use Disorders Identification Test (AUDIT) which, at a cut-off point of 8+ (score range 0–40), has a sensitivity and specificity of 92 and 94% at identifying hazardous, harmful or dependent drinking [12]. Thus, heavy drinking is both a public health problem and a major burden on the criminal justice system.

Despite a wealth of evidence supporting brief alcohol intervention at reducing heavy drinking in health services [13-15], there has been relatively little research in the criminal justice sector. A recent quasi-experimental study in eight police custody settings in England found reduced alcohol consumption amongst arrestees who received a brief intervention; however, those in the intervention group were also statistically more likely to be re-arrested by 6 months post-intervention [16], although the comparison was a retrospective group of arrestees. In addition, just 34% of participants were followed up at 12 months which limits the generalisability of these findings [16]. An even lower follow-up rate of 20% was achieved in a Scottish pilot trial in community-based criminal justice settings [17]. Brief alcohol intervention in a magistrate court setting has resulted in lower levels of injury at 12 months compared to controls [18]. Finally, a recent trial based in probation (conducted as part of the SIPS programme [19]) included over 500 offenders and found reduced drinking in all three conditions (an additive combination of feedback following screening plus a leaflet-based control, 10-min structured brief advice and 20 min of behaviour change counselling) at 6 and 12-month follow-up [19]. Despite this lack of between-group differences, those in the brief advice (36%) or counselling (38%) groups were less likely to reoffend than controls (50%) in the year following brief intervention [19]. Thus, the criminal justice setting appears to be an important opportunity for alcohol intervention work, and relevant outcomes in this context should include both alcohol consumption and offending measures.

Only a proportion of individuals who are in contact with the criminal justice system are eventually managed by probation or prison services. Many arrestees only receive a caution due to ‘minor’ offences, and in other cases, there may not be sufficient evidence to proceed to a court case and/or trial. Thus, the police custody suite setting provides an important opportunity to target individuals who may be involved in alcohol-related disorder or have underlying alcohol problems but who otherwise do not have further engagement with criminal justice services. Moreover, the police setting provides an opportunity to access and intervene with a group that is perceived to be ‘hard to reach’ in public health work, that is, (often young) males who are typically socially deprived individuals and who tend not to present to health services [20].

Key barriers to carrying out alcohol research in this setting include the busyness of custody settings, time constraints for police officers and acceptability of interventions to arrestees. However, a recent mixed-method study of screening and brief alcohol interventions in a policing context provided some evidence for the feasibility of a controlled trial [11]. Screening and brief alcohol interventions were delivered for 3 months by detention officers, during finger-printing procedures, and this input appeared to be acceptable to the majority of arrestees since 77% agreed to be screened and 98% of those screening positive consented to brief alcohol intervention [11]. Nonetheless, whilst all detention officers in one police station were successfully trained to deliver study interventions, they reported mixed views about the experience with just half the officers expressing enthusiasm for the role of delivering brief interventions in this setting [11]. Additional exploratory work by Coulton et al. reported that the majority of English offenders (74%) in police, prison and probation settings did not feel coerced into participating in screening and brief intervention activity [21]. In one sub-urban area of France, forensic physicians, described as addiction specialists, reported high rates of substance use in the police custody setting and concluded that intervention delivery was feasible because arrestees seemed responsive to brief intervention input [22]. The perspectives of the arrestees were not reported. Consequently, it is necessary to establish views from both arrestees and routine police staff about the acceptability of screening and brief alcohol interventions in this setting. In addition, work in this field needs to be scaled up to a larger number of police stations and to a wider geographical area to establish the grounds for a definitive multi-site trial.

The MRC has presented a framework for the development and evaluation of complex interventions [23]. This present work represents the development and piloting phases of this framework. Consequently, we propose a pilot feasibility trial, including a specified comparator condition, in two areas of England involving multiple police stations.

### Aim

This pilot feasibility study aims to investigate whether it is possible to recruit and retain arrestees in a cluster randomised control trial (C-RCT) of brief interventions delivered by detention officers or drug and alcohol workers aimed at reducing risky drinking in arrestees who are detained and managed in a custody suite setting. The specific objectives of this pilot trial are as follows:

- To estimate the parameters for the design of a definitive C-RCT of alcohol screening and brief intervention, including rates of eligibility, consent, participation, retention at 6 and 12 months and the data completion of the outcome measures
- To explore the feasibility and acceptability of alcohol screening and brief intervention and trial processes from the perspectives of custody suite staff and arrestees
- To explore the fidelity of brief intervention delivery in this setting
- To pilot the collection of cost and resource use data to inform the design of cost-effectiveness/utility analysis in a definitive trial
- If success criteria are met, to develop the protocol for a definitive C-RCT and economic evaluation of the impact of alcohol screening and brief interventions compared to standard advice to reduce alcohol consumption.

Success criteria for this pilot trial would be to successfully recruit and deliver interventions to 60 arrestees per condition over 6 months and follow up at least 50% of them at 23 months across the two geographical study areas (see ‘Intervention 1—brief advice’ below). Further, that qualitative data indicate that trial processes are acceptable to staff and arrestees within this setting.

## Methods/design

### Setting

Nine custody suite sites across four police forces and two geographical areas (North East and South West England) will be recruited into the study. Three police forces will be situated in the North East and one in the South West.

### Interventions

The three arms of the trial are additive and will include screening only (control group), brief advice (intervention 1) on the same occasion and brief lifestyle counselling (intervention 2) on a subsequent occasion.

#### Control condition

Arrestees will be screened using the AUDIT and assessed via the baseline questionnaire but they will not receive further alcohol-specific information or feedback on their screening responses.

#### Intervention 1—brief advice

Arrestees will be screened and assessed and then personalised feedback will be given on their screening score. This will then be followed by 10 min of structured brief advice about alcohol and its impact on health and offending behaviour. The brief intervention procedures will be fully manualised and based on the ‘How much is too much’ brief intervention programme (level 1), which was highlighted by the National Institute for Health and Clinical Excellence alcohol prevention guidance (PH24) as an evidence based brief alcohol intervention programme [24].

#### Intervention 2—brief lifestyle counselling

Arrestees will be screened and assessed and receive personalised feedback on their screening score. This will then be followed by 10 min of brief advice (as above) delivered by the individual carrying out the screening. Arrestees will then also be invited to a subsequent 20-min session of behaviour change counselling within 1 month and at an appropriate venue taking into consideration confidentiality, risk assessment and participant convenience and comfort. The counselling will be based on the ‘How much is too much’ programme (level 2) and include assessment of readiness to change (via numeric scales considering both the importance of, and confidence about, changing drinking behaviour), exploration of the pros and cons of changing and development of a practical and personalised six-step plan for changing drinking habits.

### Participants—interventionists

In two of three North East police forces, screening and brief advice will be carried out by detention officers. Detention officers have a distinct, civilian role within the custody suite setting and are responsible for an arrestee’s well-being; they have no role in interviewing or charging an arrestee. Therefore, these staff were previously identified as a suitable professional group to deliver the screening and brief interventions [11]. Furthermore, detention officers fingerprint arrestees immediately before they are released which represents an opportunity to screen and intervene at a time when the arrestee is least likely to be influenced by any alcohol consumption and has had time to reflect on the arrest incident [11]. However, early developmental work for this study indicated that the role of the detention officer had become privatised in some force areas, meaning that additional duties could not be added to their existing contracts. In these areas, drug and alcohol workers routinely support arrestees from a well-being perspective. Consequently, in the third North East site and in the South West, drug and alcohol workers carried out the screening and brief advice instead of detention officers. All staff will receive the same training in screening and brief advice procedures.

In all North East custody suites, a trained alcohol health worker will deliver the brief lifestyle counselling part of intervention 2. In the South West, this same individual will train the drug and alcohol workers to deliver the behaviour change counselling, meaning the same individual will deliver all parts of the intervention, unlike in the North East (see below).

### Participants—arrestees

Participants in this trial will be individuals aged 18 or above who are arrested (for any reason) and brought into the police custody suite to be either charged or released. Universal screening is more effective than targeting specific offences at identifying the full range of arrestees who could benefit from brief alcohol intervention because drinking level (or pattern) is not always known or recorded at the time of an offence. It is the subsequent screening outcome that will then determine if brief alcohol intervention should be delivered or not. The inclusion of hazardous and harmful, as well as dependent, drinking fits with the wider literature in this field [13-15] which indicates that the former groups often show greater responsiveness in terms of behaviour change than the latter. Thus, focusing on this spectrum of drinking has the greatest public health impact.

### Screening

Following informed verbal consent, screening will be carried out by participating detention officers or drug and alcohol workers using the 10-question Alcohol Use Disorders Identification Test (AUDIT) [12]. Based on alcohol consumption during the previous 6 months, participants who score 8+ on the AUDIT (score range 0–40) will be eligible for the trial and asked to provide informed written consent. A score of 8+ indicates hazardous (score of 8–15), harmful drinking (16–19) or probable dependent drinking (20+) [12]. It is anticipated that screening will take place during routine processing, whilst fingerprints and other details are taken in the North East or during assessment in the South West. Routine processing is the last procedure to take place before an arrestee leaves custody; this means arrestees have to be suitably fit to be released and will be sober when screening takes place.

#### Inclusion criteria

The first part of the screening process is an assessment of eligibility for the study and particularly whether or not an arrestee has the capacity to understand what they are being asked to consent to. Arrestees who are not deemed to be a danger to themselves or police staff will be eligible to take part in the trial if they can speak, read and write English and have a fixed abode and score 8 or more on the AUDIT questionnaire.

#### Exclusion criteria

An arrestee will not be eligible if they are grossly unwell (i.e. seeking medical attention), including having major psychiatric problems or alcohol withdrawal suggesting dependence which would require referral to specialist care. Major psychiatric problems include any serious mental health problems such as severe learning difficulties or schizophrenia. Any arrestee currently involved in other alcohol research will also be excluded from the study.

### Randomisation

The study will employ a cluster randomisation design in order to avoid contamination. Detention officers and/or drug and alcohol workers will be randomised to one of three trial arms using block randomization. The cluster randomisation involves randomising workers at each site to a trial arm, without allowance for whether the worker is a detention officer or a drug and alcohol worker. Allocation will be conducted by the study statistician. The randomisation sequence will be created in Stata 12 [25], using the macro *sxd1_2* (1) that permits block randomisation. The randomisation will be stratified by police custody site, with equal probabilities for the three arms and randomly generated varying block size. The trial statistician will write the Stata do file to perform the randomisation, but the randomisation sequence will be generated by another statistician not involved in the study to ensure concealment of allocation.

The arm to which each detention officer or drug and alcohol worker will be allocated will be written in a note placed within a sealed opaque envelope, with the officer’s name or unique ID number written on the outside. Neither the trial statistician nor the person delivering training to the officer will be aware of the allocation prior to the envelope being opened—in the officer’s presence—immediately before the training is delivered. Screening materials will be delivered in three separate, clearly marked boxes at each site. Each detention officer or drug and alcohol worker will be instructed to only take packs, in sequence, from the box that is labelled with the condition they have been allocated to. In each box, each individual pack will also be labelled in case of any movement due to the busy nature of the environment.

### Consent

Eligible participants (those who score 8+ on the AUDIT) will have the study explained to them by the detention officer or drug and alcohol worker and will be given an information leaflet about the study. Participants will be given as much time as they require to decide whether or not they want to take part in the study. If the arrestee decides to take part, written informed consent will be obtained. Any arrestee will be informed that they can refuse to participate without giving a reason for doing so, and anyone who has already consented can withdraw from the study at any time and does not need to give a reason for doing so. All consent forms include a box for arrestees to tick which gives their permission for the research team to look at their Police National Computer (PNC) and arrest data. All consent forms will be kept in a secure place at each police custody site until collected by a study researcher.

### Measures

The key outcomes measures in this pilot trial will be the percentage of eligible participants recruited and percent of enrolled participants retained at 12 months. Due to uncertainty about the mobility and traceability of our study population, the 6-month follow-up will enable us to re-check contact details and assess interim attrition.

In addition, we will administer a number of tools to assess response variability in key measures likely to be used in a future definitive trial. AUDIT score has been found to be responsive to change following alcohol intervention [26] and has been successfully used as an outcome measure in a recent trial with offenders [19]. Thus, AUDIT will be used to measure changes in alcohol consumption and risk status following brief intervention. In addition, we will use the modified Readiness to Change Ruler [27] to assess readiness to change drinking behaviour on a numerical scale of 0–10 and the EQ-5D to measure Quality of life [28]. We will also collect demographic data including age, gender, ethnicity and postcode, as well as the reason that the individual was arrested and subsequently whether the arrestee was charged or released without charge. Permission will be sought from participants at baseline for linkage to a) police force arrest data in order to validate self-report arrest data and b) PNC data in order to record and validate conviction and offending history and identify offences in 12 months after recruitment.

### Follow-up

Participants will be followed up by the project researchers at 6 and 12 months post-intervention. This will be by telephone or in person depending on the participant’s preference. At both follow-up points, all tools used at baseline will be repeated. At both 6 and 12 months follow-up, the AUDIT will cover the last 6 months. At both follow-up points, we will measure alcohol-related problems via the brief Alcohol Problems Questionnaire (APQ) [29,30] and collect data on health and social care resource costs via the modified short Service Use Questionnaire (S-SUQ) in preparation for future economic evaluation (UKATT [31]).

### Sample size

**Figure 1:**
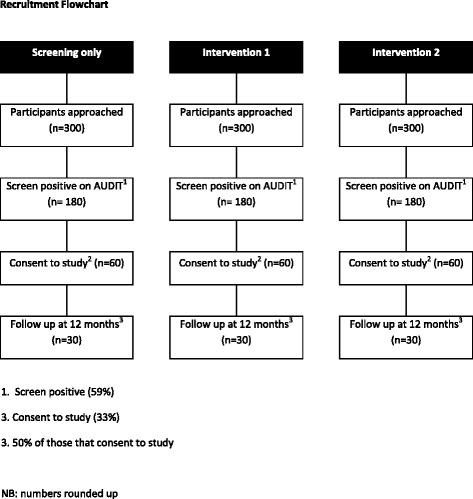
**Recruitment flowchart.**

This is a feasibility pilot trial and not an outcome evaluation; therefore, a formal power calculation is not required [32]. However, providing data that can inform the power calculation of a definitive trial is an important function of a pilot study; a minimum number of 30 participants per study arm (90 in total) at 12 months is recommended to estimate a parameter for this purpose [33] (Figure 1). Whilst this study involves cluster rather than individual randomisation, the relatively small average cluster size means that no major adjustment to the value cited by Lancaster et al. [33] is required. Specifically, assuming that around 30 detention officers or drug and alcohol workers are randomised and using an intra-cluster correlation coefficient of 0.04, the design effect would be 1 + [(90 / 30) −1] × 0.04 = 1.08.

Based on Brown et al. [11], we estimate that 59% of screened arrestees will score 8 or more on the AUDIT and of these, 33% will consent to participate in the study. We have assumed a conservative follow-up rate at 12 months of 50%. Detention officers or drug and alcohol workers in this pilot trial will need to approach 300 arrestees per arm, 900 in total. With nine police custody sites across the two geographical areas in this study, hence, each site will need to approach around 225 arrestees over a 4-month period or approximately 56 per month. Success criteria will be to successfully recruit and deliver interventions to 60 participants per condition (180 in total) and follow-up at least 50% of these individuals at 12 months (90 in total). To minimise attrition during training, detention officers and drug and alcohol workers will be encouraged to obtain detailed participant contact details (particularly telephone numbers or email addresses) to enable follow-ups to take place with minimal difficulty.

### Financial incentives

Each police custody suite will receive £1,000 to support the delivery of the study and for the burden of the research taking place. Participants in the study will be recompensed for any travel and child care costs incurred as part of the follow-up or qualitative parts of study.

### Training and support

All detention officers or drug and alcohol workers will be trained to deliver interventions by the research team, which has prior experience in the delivery of brief interventions. Training will be delivered as on-site, outreach training. Such outreach training was found to be the most cost-effective implementation strategy for alcohol screening and brief intervention delivery in other settings [34]. Detention officers and drug and alcohol workers will be trained only in the condition to which they are randomised and encouraged not to discuss what they have learned with colleagues. Training will be guided by a written manual and detention officers or drug and alcohol workers will be given a copy of the intervention manual to keep for reference. The training will last around 1 h and will be done either one on one or in a group, depending upon the custody site’s preference, given their time restraints and shift patterns (all these details will be recorded by research staff). Training in intervention 2 for those in the South West will be delivered by the experienced alcohol health worker with responsibility for conducting behaviour change counselling at North East sites and will last around 3 h. In addition to the manualised materials, there will be video-based material of the alcohol health worker delivering key elements of the behaviour change counselling. The session is intended to be highly interactive and will include exercises designed to build core skills including how to assess readiness for change via scaling questions, identify change talk in arrestees and ask open questions that identify motivation for change. The research team will support the staff in implementing study procedures through weekly contact (in person or via telephone) with each police custody suite, and paperwork relevant to the research will be provided by the research team, who will also act as the site study coordinator.

### Qualitative work to explore acceptability and intervention fidelity

A definitive study should only be conducted if the study procedures are found to be acceptable to either detention officers or drug and alcohol workers and arrestees. Thus, a qualitative sub-study will be conducted with a purposive sample of approximately 15–24 arrestees and 8–12 detention officers and/or drug and alcohol workers. In-depth interviews will be guided by a topic guide and will explore the acceptability of the study procedures. Issues relating to consent, the potential for coercion around participation, the alcohol screening process, the timing, content and manner of intervention delivery, the burden of time and how this work is best embedded alongside other activity in the custody setting will be explored with careful prompting where responses seem curtailed. We will also explore intervention fidelity by seeking specific details about the form of intervention that was due to be received or delivered and asking about key elements of the expected intervention. Finally, we will seek views on the ethical aspects of incentivising research participants in this challenging criminal justice setting and if any concerns or contradictions are perceived regarding health care in a criminal justice setting.

We will aim for a maximum variation sample to achieve a broad perspective on the issues being explored. Sampling criteria for arrestees will be age, sex, reason for arrest, study condition and ‘type of drinker’ based on AUDIT category [12]. Sampling criteria for detention officers and drug and alcohol workers will be sex, geographical area, study condition and number of arrestees approached. Emergent issues from earlier interviews will be explored in subsequent interviews and the total number of interviews will be determined by data saturation (no new issues or themes emerging from within/across participants). Interviews with detention officers and drug and alcohol workers will take place after baseline data collection has ceased and will be conducted in a place convenient to the police station. Interviews with arrestees will take place after follow-up data collection has ceased. The venue for interviews with arrestees will be negotiated taking into consideration risk assessment and arrestees convenience, confidentiality and preference. Interviews will last for approximately 45 min each. All interviews will be audio-recorded and transcribed verbatim for analysis.

### Planned analysis

#### Statistical analysis

As this is a pilot feasibility trial, no formal hypothesis is to be tested. The aim of this trial is to provide robust estimates of recruitment, retention and consent rates and provide data for the power calculation of a definitive trial. Descriptive analysis will include participant characteristics (age, sex, educational attainment, social deprivation), numbers and percentages recruited and retained at both follow up points and variability in study measures. In particular, these measures will be considered on an intention to treat basis at 6 and 12 months after the intervention has taken place.

If a definitive trial is judged to be feasible, a decision will be made on the choice of the primary outcome measure. The calculation of sample size for this trial would follow the principles described in the extension of the CONSORT 2010 statement to cluster randomised trials [35] and would take account of several factors, as follows:

- If the primary outcome measure was continuous, then its standard deviation or (if not normally distributed) the width of its interquartile range based on variability within clusters would be estimated, using the results from the pilot feasibility trial.
- Alternatively, if the primary outcome measure was dichotomous (e.g. yes/no for the presence of a characteristic), then the sample size calculation would take account of the proportion of participants with this characteristic in the pilot feasibility trial, with adjustment for differences between clusters.
- The intra-cluster correlation coefficient for the primary outcome measure would be estimated, although—since this is likely to have a large sampling error—alternative values may be used to examine the sensitivity of the calculated sample size to the choice of value.
- Experience from the pilot feasibility trial about the number of persons who might be recruited per detention officer (or drug and alcohol worker) would also be taken into account, so that a decision could be made about how many clusters are required in a definitive trial.

Health economics assessment will consider costs (training, screening arrestees, delivering interventions, practitioner time) and outcomes (reduced alcohol consumption, reduced problems, re-offending rates).

#### Qualitative analysis

Analysis will be conducted using a structured thematic approach to systematically code, classify and organise interview content into key themes, with the Framework approach employed to organise the analysis [36]. This analytic strategy is characterised by a more deductive than inductive approach and is appropriate for qualitative health research with objectives linked to an applied research question and a delimited time frame. The interview recordings will be reviewed and important or recurrent themes in interviewees’ talk will be identified. These will be combined with a list of key themes of research interest derived from the aims and questions for the study and coded within a framework of a priori headings. Finally, the descriptions of headings within the framework will be compared and the relationships between them elaborated to provide a consistent interpretation of the dataset as a whole [37].

### Ethical and research governance approval

This pilot feasibility trial is funded by NIHR School for Public Health Research and has been assigned the trial reference number ISRCTN89291046. Ethical approval has been granted by Newcastle University (Reference 00754), who will act as sponsor for the research. The trial will be managed through a central co-ordinating team. The Programme Management Group will be responsible for ensuring the appropriate, effective and timely implementation of the trial. A Trial Steering Group will be appointed and will concentrate on the progress of the trial against projected rates of recruitment and retention, adherence to the protocol, participant safety and the consideration of new information of relevance to the research question. Written charters will be agreed and used by both groups.

### Timescale

The duration of the trial is 24 months.

## Discussion

It is important to perform pilot feasibility RCTs when the logistics of a large-scale trial are unclear [32,33]. Although there is evidence of effectiveness of alcohol screening and brief intervention in primary care [14] and other health settings [38] there is little work in criminal justice settings, and specifically in a police context. The custody suite setting is different from other criminal justice settings, such as probation or prison, as many arrestees will be released without charge and are therefore not necessarily criminalised. The alcohol-related studies that have been carried out to date have low levels of follow-up and lack a concurrent comparison condition, meaning that the effectiveness of health promoting and/or crime reducing interventions cannot be ascertained [16]. The findings from this pilot feasibility study will indicate whether or not and how a definitive trial can establish the effectiveness and cost-effectiveness of alcohol screening and brief intervention in a police custody suite setting.

A key consideration in the design of a potential future trial is the most appropriate level of randomization. The decision to randomise at the level of the detention officer/drug and alcohol worker in this pilot trial was based on two criteria: 1. the need to minimise the risk of contamination between intervention conditions and 2. the need to maximise research efficiency due to limited study resources. In the former situation, staff specifically trained to deliver brief intervention would be no longer able to deliver usual care due to likely skill enhancement and so individual randomization (at the level of arrestees) would not be possible. In the latter situation, there has been a trend towards the development of larger, centralised custody suites in England where a growing number of detention staff manage multiple arrestees in a single (often larger town or city-centre) location and we only had the resources to recruit, train and support staff in a limited number of areas. Nevertheless, detention officers/drug and alcohol workers are allocated specific arrestees to manage and remain their point of contact and care throughout the detention episode. Thus, randomization at the level of the detention staff enabled each one to deliver input according to condition-specific training and their own intervention materials whilst allowing recruitment of several staff members in a single custody suite site, thus reducing sample size needed for the trial. There remains a small risk of potential contamination through discussion of the study amongst detention staff, although senior managers had indicated that this was likely to be minimal due to the level of busyness within suites and also specific shift rotas. Nevertheless, the latter issue will be explored during the planned qualitative interview work.

Exploring the views of relevant staff and arrestees will also be essential to inform whether the police custody suite setting is an acceptable context for public health intervention. These data will also provide a broader understanding of how screening and brief interventions are actually delivered and received in the custody suite. Thus, we can clarify if we have the attitudinal support and ideal processes in place for a future trial. If recruitment and retention success criteria are also reached, these findings will be used to develop a protocol for a definitive trial, with a sample size calculation which will usefully extend the evidence base in this field at an international level.

### Trial status

The trial is currently recruiting.

## Abbreviations

APQ: Alcohol Problem Questionnaire
AUDIT: Alcohol Use Disorder Identification Test
C-RCT: Cluster randomised controlled trial
NIHR: National Institute of Health Research
S-SUQ: Short Service Use Questionnaire
